# Monocyte-Derived cxcl12 Guides a Directional Migration of Blood Vessels in Zebrafish

**DOI:** 10.1161/ATVBAHA.124.321588

**Published:** 2025-01-23

**Authors:** Xiaofeng Lu, Xiaoning Wang, Bowen Li, Xin Wang, Xuchu Duan, Dong Liu

**Affiliations:** 1 School of Life Science, Nantong Laboratory of Development and Diseases and Co-Innovation Center of Neuroregeneration, Nantong University, China.

**Keywords:** animal fins, blood vessels, cues, monocytes, zebrafish

## Abstract

**BACKGROUND:**

Sprouting blood vessels, reaching the aimed location, and establishing the proper connections are vital for building vascular networks. Such biological processes are subject to precise molecular regulation. So far, the mechanistic insights into understanding how blood vessels grow to the correct position are limited. In particular, the guide cues and the signaling-originating cells remain elusive.

**METHODS:**

Live imaging analysis was used to observe the vascular developmental process of zebrafish. Whole-mount in situ hybridization and fluorescent in situ hybridization were used to detect the expression profiles of the genes. Single-cell sequencing analysis was conducted to identify the guiding protein and its originating cells.

**RESULTS:**

Taking advantage of live imaging analysis, we described a directional blood vessel migration in the vascularization process of zebrafish pectoral fins. We demonstrated that pectoral fin vessel c migrated over long distances and was anastomosed with the second pair of intersegmental vessels. Furthermore, we found the cxcl12a-cxcr4a axis specifically guided this long-distance extension of pectoral fin vessel c–intersegmental vessel, and either inhibition or overexpression of cxcl12a-cxcr4a signaling both mislead the growth of pectoral fin vessel c to ectopic areas. Finally, based on an analysis of single-cell sequencing data, we revealed that a population of monocytes expresses the Cxcl12a, which guides the migration of the vascular sprout.

**CONCLUSIONS:**

Our study identified Cxcl12a as the signaling molecule for orchestrating the organotypic-specific long-distance migration and anastomosis of the pectoral fin vessel and the intersegmental vessels in zebrafish. We discovered a specific cluster of *gata1* (globin transcription factor 1)-positive monocytes responsible for expressing Cxcl12a. The findings offer novel insights into the mechanisms underlying organotypic vascularization in vertebrates.

**Highlights**

- We found a directed migration of angiogenic sprouts in the zebrafish pectoral fin vascular development process.
- We demonstrated the specific elongation and connection of the pectoral fin vessel c to the second pair of intersegmental vessels.
- The Cxcl12/Cxcr4 axis was identified as the critical guidance for this long distance–directed migration.
- Based on the analysis of single-cell sequencing data and cell ablation experiments, it was revealed that a population of monocytes expresses Cxcl12a involved in this process.

As one of the earliest organs of vertebrates, vascular networks are essential for providing oxygen, transmitting signals to other organs, and transporting circulating metabolites and wastes.^1^ A vascular network’s correct establishment is a prerequisite for its function. Misconnection of blood vessels can lead to diseases such as arteriovenous malformations.^2^ Blood vessels are formed in 2 sequential ways: vasculogenesis and angiogenesis. While vasculogenesis mainly occurs at the early developmental stages as the de novo formation of vessels based on angioblasts, angiogenesis is a process to generate new blood vessels from the preexisting ones, which involve several angiogenic events, including sprouting, elongation, anastomosis, and pruning.^3–5^

**See cover image**

In sprouting angiogenesis, anastomosis occurs when the new capillaries from parental vessels fuse with other sprouts or preexisting vessels. Anastomosis is a fundamental and essential process for building vascular networks; it is guided by tip cells and involves other cellular behaviors, including sprouting, migration, adhesion, and lumen formation, which thereby confer the anastomosis complex cellular and molecular mechanisms that are still poorly understood.^6^ So far, only VE-cadherin has been validated to be responsible for vascular fusion, and this anastomosis is caused by filopodial contacts between 2 proximal tip cells as an initial trigger.^7^ Studies on the directional migration and connection of 2 distant vessels have yet to be reported, and the molecular mechanisms of blood vessel migration and anastomosis still require more investigation.

In recent years, genetic approaches have facilitated research seeking vascularization-associated functional genes. However, the studies are still hampered by the inability to observe the vascular developmental process in vivo, especially in mice. Zebrafish are transparent at the embryonic and juvenile stages, which allows us to observe the vascular developmental process in specific tissues and organs by live-imaging analysis.^3,8,9^ The dorsal longitudinal anastomotic vessels of zebrafish formed by anastomosis of neighboring segmental arteries are particularly suitable for investigating the underlying cellular and molecular mechanisms of anastomosis.^10,11^

So far, little has been known regarding the regulating mechanisms of directional blood vessel migration and connection, especially the involved guide cues and the signaling-originating cells. Chemokines are a family of chemotactic cytokines that play indispensable roles in vascular development. The CXCL12 (C-X-C motif chemokine ligand 12)/CXCR4 (C-X-C motif chemokine receptor 4) axis has been validated to be essential for vasculature formation in diverse organs such as the kidney and the gastrointestinal tract.^12,13^ Here, we used different transgenic zebrafish lines to study the organotypic vascular development in pectoral fins, which are pretty transparent during the lifespan. We observed the specific long-distance migration of the pectoral fin vessel (PFV) and the subsequent anastomosis with the second pair of intersegmental vessels (ISVs). We further validated that the monocyte-derived CXCL12a (C-X-C motif chemokine ligand 12 a) was essential for guiding this process via the CXCL12a/CXCR4a (C-X-C motif chemokine receptor 4a) axis. Our research provided new insights into the blood vessel formation during the embryonic development of zebrafish.

## Materials and Methods

### Data Availability

The authors declare that all supporting data are available within the article and its Supplemental Material. The single-cell RNA-sequencing data (GSE178150) used in this study can be accessed in the public database Gene Expression Omnibus (https://www.ncbi.nlm.nih.gov/geo/).

### Ethics Statement

All animal experimentation was performed in accordance with the National Institutes of Health guidelines for the care and use of laboratory animals (http://oacu.od.nih.gov/regs/index.htm) and ethically approved by the Administration Committee of Experimental Animals, Jiangsu Province, China (approval ID: SYXK(SU) 2017–00121).

### Zebrafish Housing and Feeding Conditions

Zebrafish embryos and adults were raised using the methods described previously.^14^ Embryos were obtained through natural mating and maintained at 28.5 °C. The stages of zebrafish embryos are defined as described previously.^14^ Embryos were treated with 0.2 mmol/L 1-phenyl-2-thiourea (PTU; Sigma; P7629) to block pigmentation for further imaging analysis.

### Zebrafish Lines and Morpholino

AB was used as wildtype. The *Tg (fli1aEP:EGFP-CAAX*)^*ntu666*^ zebrafish embryos were utilized to visualize the PFV formation process. The double transgenic *Tg (flt1:*^*BAC*^*YFP::kdrl:Ras-mCherry*) zebrafish embryos were utilized to distinguish the arterial and venous vessels. *Tg (lyve1b:Topza-YFP*) zebrafish embryos were utilized to mark venous vessels during the primary angiogenesis process. *Tg(tp1:EGFP*) zebrafish embryos labeled Notch-responsive cells. *Tg(gata1: Dsred*) zebrafish embryos were used to determine the direction of blood flow.

Morpholino for *cxcr4* was used as previously reported.^15–17^ 0.3 ng *cxcr4a* ATG-morpholino (AGACGATGTGTTCGTAATAAGCCAT) was injected into *Tg(fli1aEP:EGFP-CAAX*)^*ntu666*^ 1-cell stage embryos and then incubated in E3 solution at 28.5 °C. The efficiency of the *cxcr4a* morpholino was validated by coinjection of cxcr4a-mCherry mRNA and *cxcr4a* ATG-morpholino. Embryos from each treatment were allocated randomly and transferred into 24-well plates at 20 hours post-fertilization (hpf) and incubated with PTU at 28.5 °C for imaging at 72 hpf. *Cxcl12a* mutants with 4 bp deleted in exon 1 were obtained from the China Zebrafish Resource Center.

### Generation of Genic *cxcr4a* Crispant Zebrafish

gRNA-targeted exon 2 of cxcr4a was designed (CAGCTCTGAATTCGGCTCGG) and synthesized. The crispant zebrafish were generated by injecting *cxcr4a* gRNA (0.3 ng) with Cas9 mRNA (0.9 ng) into *Tg (fli1aEP:EGFP-CAAX*)^*ntu666*^ 1-cell stage embryos and then incubated in E3 solution at 28.5 °C. Ten embryos were selected Arandomly at 24 hpf for efficiency evaluation (cxcr4a-KO-ident-F: CTTTTTCAGCACATCGTCTTTG; cxcr4a-KO-ident-R: CAGAGTGAGCACAAACAGAAGG); other embryos were transferred into PTU and incubated at 28.5 °C for imaging at 72 hpf.

### Inhibitor Treatment of LY411575 and AMD3100

The zebrafish embryos of *Tg(tp1:GFP::kdrl:Ras-mCherry*) were incubated with 2 and 5 μmol/L LY411575 (Sigma; SML0506) to inhibit Notch signal from 48 and 60 hpf separately and image at 72 hpf. Equivalent solutions of dimethyl sulfoxide were used as solvent controls. Treatment of AMD3100 was performed as previously reported.^18^ 3 nL of 300 μmol/L solution of AMD3100 (MCE; HY-10046) was injected into yolk of 36 hpf larvae of *Tg(fli1aEP:EGFP-CAAX*)^*ntu666*^ and incubated at 28.5 °C with PTU for imaging at 72 hpf.

### Whole-Mount In Situ Hybridization and Fluorescent In Situ Hybridization

Whole-mount in situ hybridization (WISH) and the preparation of RNA probes were performed as described in the previous report.^19,20^ The *cxcr4a*, *cxcl12a*, *cxcl12b,* and other cDNA fragments were cloned with the specific primers listed below using a wild-type embryo (AB) cDNA library. Digoxigenin-labeled antisense probes were synthesized using an in vitro DIG-RNA labeling transcription kit (Roche; 11175025910) with linearized pGEM-T easy vector (Promga; A1360) with each corresponding gene fragment as the template. Zebrafish embryos and larvae were fixed with 4% paraformaldehyde overnight at 4 °C, then dehydrated with gradients of methanol and stored at −20 °C in 100% methanol for subsequent analysis. The hybridization result was detected with anti-DIG-AP antibody (1:2000; Roche; 11093274910) and NBT/BCIP (1:500; Roche; 11681451001). Fluorescein RNA Labeling Mix (10×; Roche; 11685619910) was used for in vitro cxcl12a probe transcription for fluorescent in situ hybridization (FISH).

cxcr4a-ISH-F: TTTCTCCCAACGGTGTACGG

cxcr4a-ISH-R: AGATCCATTTCTGCAGCCCC

cxcl12a-ISH-F: CCGATTTCCAACGCCAAGC

cxcl12a-ISH-R: CACGACAAACACGGAGCAAAC

cxcl12b-ISH-F: GCAATATTCGCTCTTTGGGCAAAC

cxcl12b-ISH-R: AAGGTTGGTAGGCTTAGCGG

dll4-ISH-F: CGATCTGTCTGGAGGGATGC

dll4-ISH-R: TCCATTGTCCTTCTCGTGGC

flt1-ISH-F: ACATCACAGATAGCCAGCGG

flt1-ISH-R: GGGGATTGTAAGGACGCTGT

dab2-ISH-F: CAGAGGGCCAGCATCAGTTT

dab2-ISH-R: CGTCGCTGAAGGGATCTGAA

### Generation of Zebrafish Overexpressing *Cxcl12a*

The coding sequence of zebrafish *cxcl12a* was amplified from the cDNA library that was established from wild-type embryos using the specific primers of *cxcl12a*-F: CGGACGCGTGCCACCATGGATCTCAAAGTGATCGT and *cxcl12a*-R: CGACCGGTGACCTGCTGCTGTTGGGCTT, then digested with *Mlu* I and *Age* I (NEB, R3198V, R3552S) and ligated into the hsp70-MCS-mCherry vector to generate the expression plasmid *hsp70:cxcl12a-mCherry*. The transgenic zebrafish were generated by injecting *hsp70:cxcl12a-mCherry* plasmid (0.3 ng) with transposase mRNA (Tol2; 0.4 ng) into *Tg (fli1aEP:EGFP-CAAX*)^*ntu666*^ at 1-cell stage embryos and then incubated in E3 solution at 28.5 °C. For heat shock, embryos were selected randomly at 24 hpf and transferred into a 1.5-mL tube. The selected embryos were immersed in a 37 °C water bath for 1 hour for heat shock and back into 28.5 °C PTU for imaging at 72 hpf.

### NTR Depletion

The NTR (nitroreductase) depletion experiments were performed as described in the previous report.^21^ Briefly, double transgenic *Tg (fli1aEP:EGFP-CAAX*)^*ntu666*^×*Tg(mpeg1.1: NTR-mCherry*) zebrafish embryos were used to perform the NTR depletion experiment. The embryos were transferred into the medium with 10 mmol/L metronidazole (Sigma; M1547) at 36 hpf and imaged at 72 hpf.

### Imaging

Fluorescent imaging was performed using a Nikon A1R confocal microscope. Embryos were mounted in 0.7% low melting point agarose (Invitrogen; 16520100) in 1×PBS and 0.01% tricaine. All images and movies were processed and exported with NIS-Elements AR (5.10.00 for 64 bit). For time-lapse analyses, the embryos were mounted as described above. Images were obtained every 15 minutes. Images were exported as movies using NIS-Elements AR (5.10.00 for 64 bit) with 4 nm per step.

### Single-Cell Gene Expression Profile Analysis

The single-cell RNA-sequencing data used in this study (GSE178150) were obtained from the GEO database (https://www.ncbi.nlm.nih.gov/geo/). Several criteria were applied to select the single cells, including only keeping the genes that are expressed (Unique Molecular Indentifier >0) at least in 3 single cells, selecting single cells with the number of expressed genes at the range between 500 and 3000, and requiring the percentage of sequencing reads on mitochondrial genome is <5%. After normalization, cells expressing *kdrl* and *gata1a* were sorted separately. The clustering of single cells and the marker genes in each cluster were analyzed by Seurat 4.0 (https://satijalab.org/seurat/install.html).^22^ ClusterProfiler was used to do the GO enrichment analysis based on the marker genes of each cell cluster.^23^ The analyses were performed based on R4.2.2.

### Statistical Analysis

Statistical analysis was performed using a χ^2^ test; *P*<0.05 was considered statistically significant.

## Results

### Specific Anastomosis Between PFVc and the Second Pair of ISVs in the Pectoral Fin of Zebrafish

**Figure 1.**
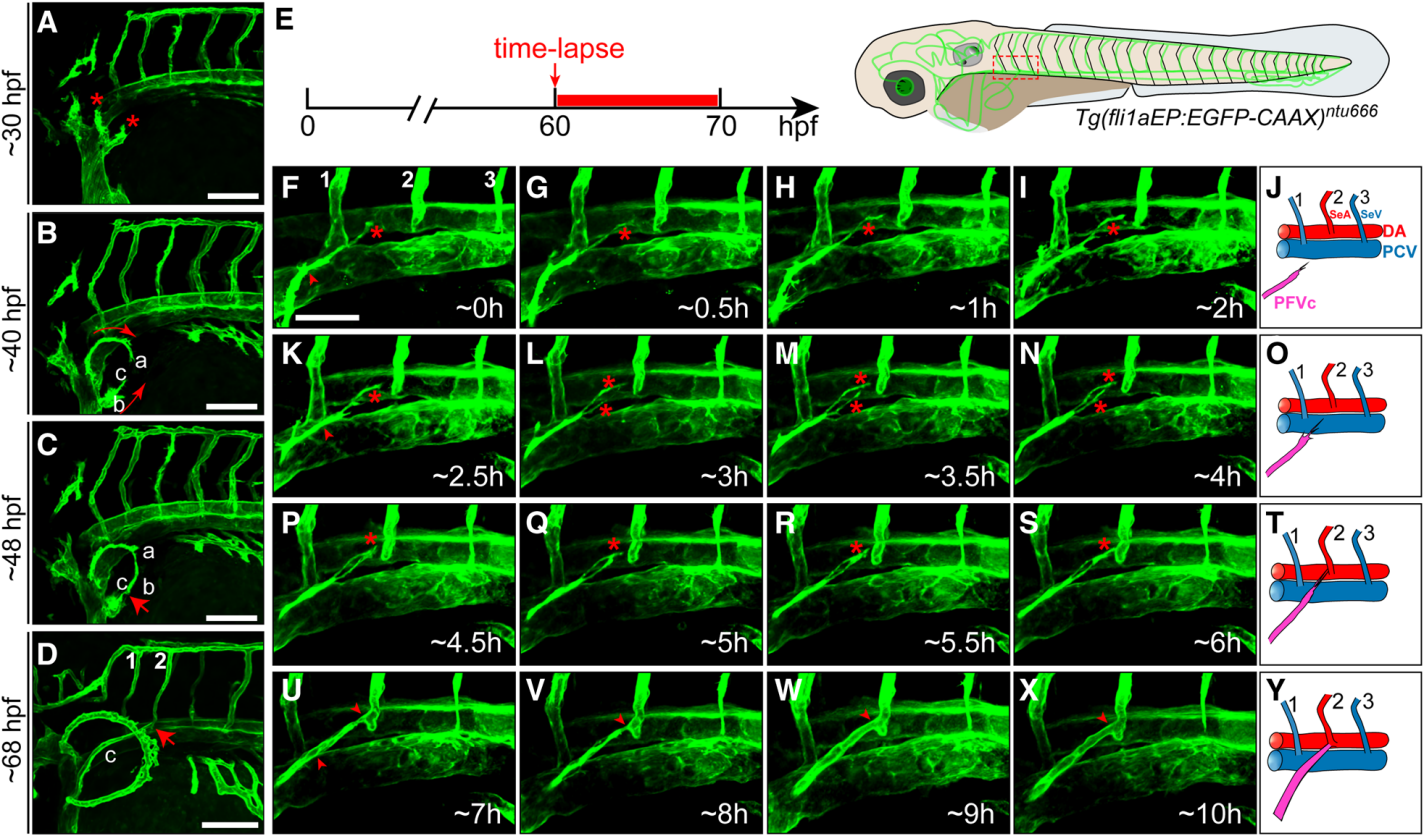
**Formation of circumferential blood vessel loop (CBVL) and anastomosis between pectoral fin vessel (PFV) c and the second pair of intersegmental vessels (ISVs). A**, Sprouts from 2 points of common cardinal vein (CCV) toward different directions. The asterisks mark the 2 initial sprouts of pectoral fin vessel (PFV) from CCV. **B**, Growth of PFVa, PFVb, and PFVc. The arrowheads show the growing directions of PFVa and PFVb. **C**, Anastomosis between PFVa and PFVb and formation of CBVL. The arrowhead points to the anastomotic point. **D**, Anastomosis between PFVc and the second pair of ISVs. The red arrowhead points to the anastomotic point. **E** through **Y**, Live-imaging analysis of the long-distance anastomotic process of PFVc with the second pair of ISVs on *Tg(fli1aEP:EGFP-CAAX*)^*ntu666*^ transgenic zebrafish. **E**, Schematic diagram showing the imaging time window and area. **F** through **I**, Growth of PFVc toward the trunk. The asterisk marks the target ISV-directed filopodia. At the early stage, >1 filopodia sprouted from CBVL and grew in different directions. The arrowhead points to the other filopodia. **K** through **N**, The filopodia was guided to the second pair of ISVs. During this stage, only the filopodia oriented to the second pair of ISVs could extend and reach the target while the others all regressed. **P** through **S**, The connection of PFVa with the target ISVs. **U** through **X**, The lumenization process of PFVc-ISVs. **J**, **O**, **T**, and **Y**, Schematic diagram of the anastomotic process. The experiments were executed in triplicate. Scale bars=50 µm. DA indicates dorsal aorta; hpf, hours post-fertilization; PCV, posterior cardinal vein; SeA, segmental vessel artery; and SeV, segmental vessel vein.

In the previous study, Yano et al^24^ described the process of PFV development in zebrafish, including that 2 blood vessels from the anterior and posterior sides of the fin started growing at 43 hpf and then connected to form the circumferential blood vessel loop (CBVL) at 48 hpf. Here, we observed the whole angiogenic process in the pectoral fin of zebrafish embryos. We found something interesting: the PFVc grew progressively along the outer surface of the yolk toward the dorsal direction, bypassed the first pair of ISVs, and finally reached and fused with the proximal end of the second pair of ISVs at 68 hpf (Figure 1A through 1D; Figure S1; Video S1). To validate the specificity of the connection between PFVc and the second pair of ISVs, time-lapse imaging analysis was performed to observe the anastomotic process (Figure 1E). The results reconfirmed the process that the PFVc grew toward the trunk and fused with the second pair of ISVs. Interestingly, during this process, although several filopodia were growing toward different directions at the beginning, only the filopodia toward the second pair of ISVs could successfully extend and fuse with the target vessels; other ones toward the other directions all degenerated and eventually disappeared (Figure 1F through 1Y; Video S2). The results indicated that the PFVc was specifically directed for long-distance anastomosis with the second pair of ISVs.

### Arterial-Venous Identification of CBVL

**Figure 2.**
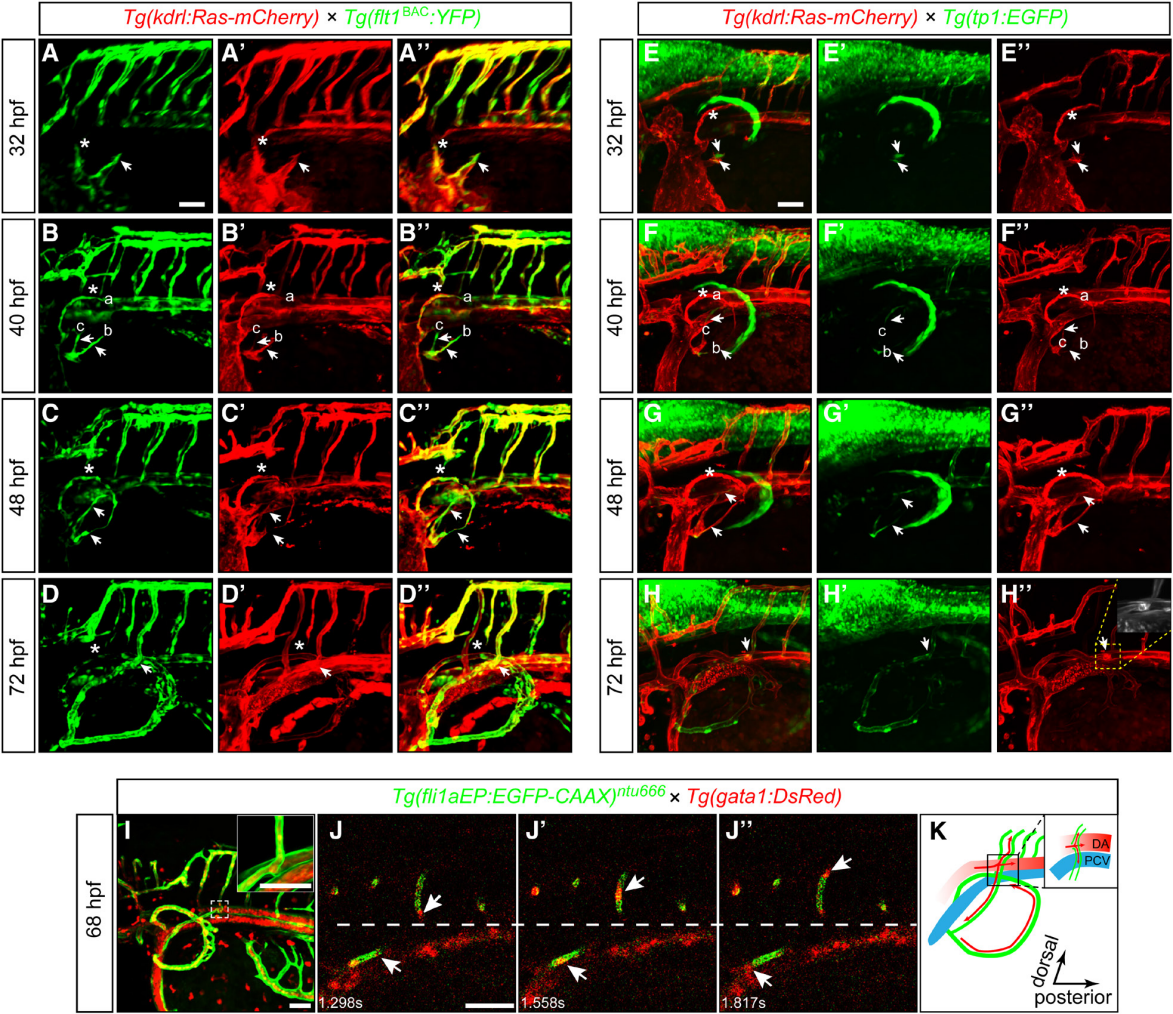
**Arterial-venous specification and blood flow direction of pectoral fin vessel (PFV) c. A** through **D″**, Arterial-venous specification of circumferential blood vessel loop (CBVL) during the development of PFV, the asterisks mark the blood vessels sprouted from the dorsal side of the common cardinal vein (CCV), and the arrows mark the blood vessels sprouted from the ventral side of CCV. **A** through **A″**, The vessels sprouted from CCV were not differentiated at 32 hours post-fertilization (hpf). **B** through **B″**, At 40 hpf, the dorsal sproutings (PFVa, marked with asterisks) grew faster than the ventral ones, which branched into 2 new vessels (PFVb and PFVc). **C** through **C″**, At 48 hpf, before perfusion, the PFVb and PFVc developed into an artery, while the PFVa developed into a vein indicated by the expression of *flt1* in green fluorescence. **D** through **D″**, After perfusion, the specification of CBVL was confirmed. **E** through **H″**, Notch signal mediated the artery development of pectoral fin vessels. **E** through **E″**, At 32 hpf, green fluorescence was enriched in the sporting PFVb and PFVc but not PFVa. **F** through **G″**, Green fluorescence was observed at the tip cells of PFVb and PFVc, while no signal was detected along PFVa at 40 and 48 hpf. **H** through **H″**, The formation and specification process of CBVL confirmed the arterial property of PFVc and the second pair of intersegmental vessels (ISVs) after anastomosis and perfusion of the vessels. **I** through **J″**, The direction of blood flow in PFVc and its target ISVs. **J** through **J″**, Side view of time-lapse imaging in the white dashed box in **I**, arrows indicate the blood cells. **K**, Schematic diagram of the direction of blood flow in PFVc and its target ISVs. The experiments were executed in triplicate. Scale bars=50 µm. DA indicates dorsal aorta; and PCV, posterior cardinal vein.

To further confirm the specification of the CBVL vessel, the double transgenic zebrafish line *Tg(kdrl:Ras-mCherry::flt1:*^*BAC*^*YFP*) was used to analyze the arterial-venous identity. The transgenic line labels the endothelial and arterial endothelial cells with red (*kdrl:Ras-mCherry*) and yellow (*flt1:*^*BAC*^*YFP*) fluorescence, respectively. The results showed that at the budding stage at 32 hpf, the yellow fluorescent dots were evenly distributed in PFVa, PFVb, and PFVc, indicating that these new vessels were unspecified (Figure 2A through 2A″). However, from around 40 hpf, the PFVb and PFVc started to express higher levels of *flt1* than PFVa did, indicating that PFVb and PFVc were becoming arteries and PFVa tended to be vein (Figure 2B through 2C″). Concurrently, PFVa continuously express both flt1 and Ras from 40 to 48 hpf. After the fusion between PFVc and the ISVs, and perfusion of the CBVL, green fluorescence in PFVa gradually diminished, leaving only red fluorescence at 72 hpf, indicating the venous characteristic of PFVa. Consequently, the border between the artery and vein in the CBVL was more apparent at 72 hpf (Figure 2D through 2D″). The result was further validated by using another transgenic line *Tg(lyve1b:Topza-YFP::kdrl:RAS-mCherry*), which labels the venous cells and endothelial cells at the angiogenesis stage (Figure S2). In addition, we performed ISH experiment to detect the expression of *dll4, flt1*, and *dab2*, which specifically label the arterial and venous cells, respectively. Expression of *dll4* and *flt1* could be detected at PFVb and PFVc at 48 hpf, proving both the PFVb and PFVc were arteries, while *dab2* was highly expressed at PFVa at 60 hpf, indicating the venous property of PFVa (Figure S3).

Considering that the Notch signaling pathway plays an important role in angiogenesis via regulating artery formation.^25^ To examine the function of Notch signaling in pectoral fin vascular formation and specification, the Notch reporter line *Tg(kdrl:Ras-mCherry::tp1:EGFP*)^18^ was used to trace the vessel development and artery formation of CBVL. We observed GFP fluorescence in PFVb and PFVc since the budding stage at 32 hpf, while not in PFVa (Figure 2E through 2E″). During 40 to 48 hpf, GFP was continuously expressed in PFVb and PFVc, especially the area of endothelial tip cells, while not detected in PFVa (Figure 2F through 2G″). The results indicated different properties of PFVa from PFVb and PFVc. At 72 hpf, GFP fluorescence was clearly distributed in PFVb, PFVc, and the second ISV (arterial ISV), indicating their arterial property (Figure 2H through 2H″). Subsequently, we inhibited Notch signaling using the inhibitor LY411575 to assess the effects on vascular development. We found that the growth of PFVc was immediately inhibited when the embryos were treated by LY411575, making it unable to reach and anastomose with the target ISVs, indicating the essential role of Notch signaling in PFVc development (Figure S4).

In addition, we also observed that after perfusion, the erythrocytes moved from the dorsal aorta (DA) to the second pair of ISVs and the PFVc, confirming the artery identity of PFVc and the second ISV (Figure 2I through 2K; Video S3). Taken together, our findings revealed the process of formation and specification of CBVL in zebrafish embryos, which suggested that the arterial-venous specification of CBVL occurred before vascular connection and perfusion.

### *cxcl12a/cxcr4a* Axis Regulated the Directional Migration of the PFVc and the Anastomosis With the Target ISVs

**Figure 3.**
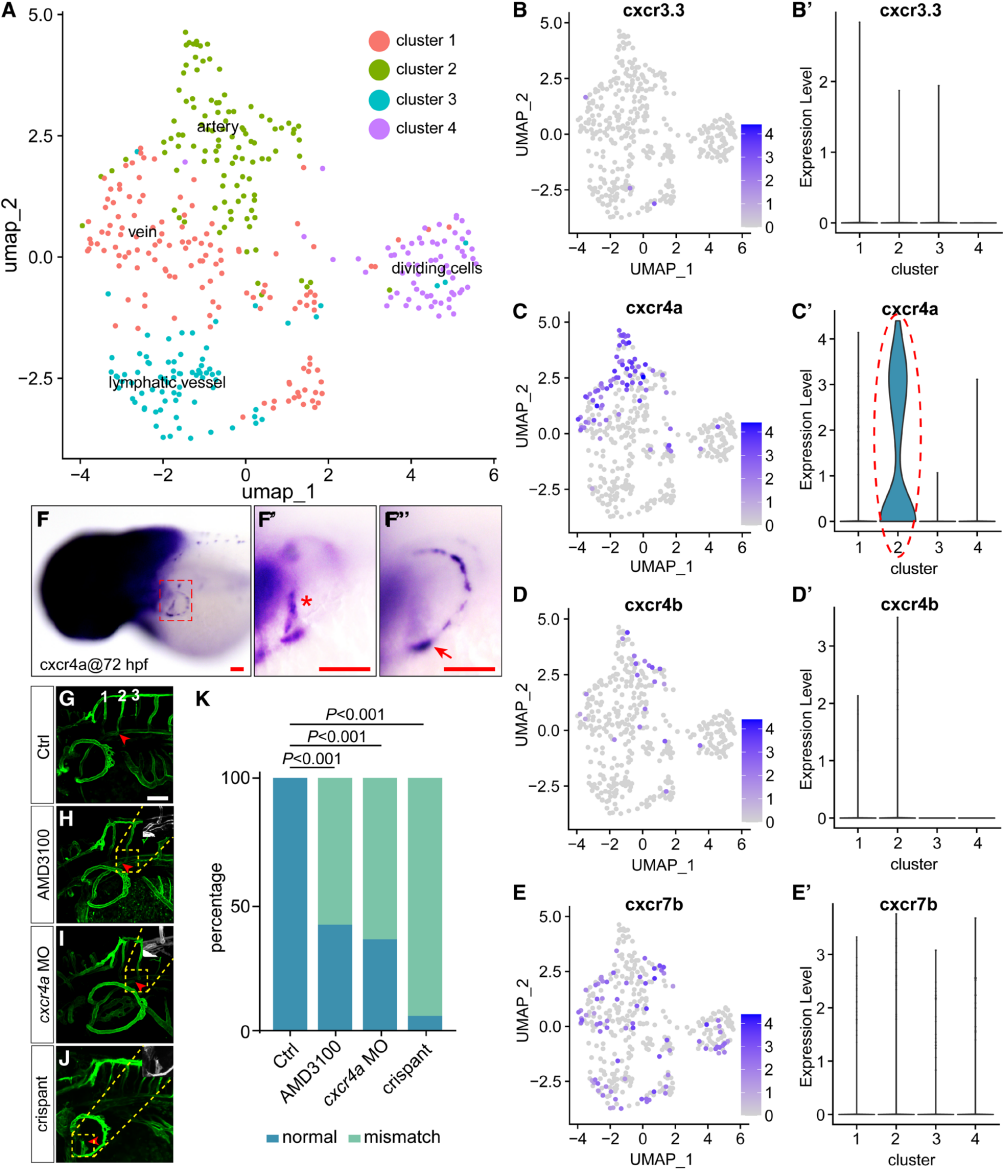
***cxcr4a* is involved in the regulation of pectoral fin vessel (PFV) c–intersegmental vessel anastomosis. A**, Clustering result of *fli1a*-positive cells (data from GSE178150) by Uniform Manifold Approximation and Projection (UMAP). **B** through **E′**, Feature plot and violin plot showing the expression of *ccxcr3.3*, *cxcr4a*, *ccxcr4b*, and *ackr3b* in the 4 clusters in **A**. **F** through **F″**, Whole-mount in situ hybridization (WISH) analysis of *cxcr4a* expression in PFVs. **G**, Live-imaging analysis of the long-distance anastomosis between PFVc and ISVs on *Tg (fli1aEP:EGFP-CAAX*)^*ntu666*^ transgenic zebrafish. **H**, AMD3100 treatment led to the misconnection of PFVc-ISVs. **I**, Knockdown of *cxcr4a* by a morpholino (MO) led to the misconnection of PFVc-ISVs. **J**, *cxcr4a* knockout crispant constructed by the CRISPR/Cas9 (clustered regularly interspaced short palindromic repeats/clustered regularly interspaced short palindromic repeat–associated 9) technique showed the loss of PFVc. **K**, The ratio of misconnection caused by the injection of AMD3100, knockdown of *cxcr4a*, and crispant knockout of *cxcr4a*. The experiments were repeated 4×; 5 embryos were used in the control (ctrl), AMD3100-treated, *cxcr4a* MO, and *cxcr4a* crispant groups for each experiment. Scale bars=100 µm. hpf indicates hours post-fertilization.

The finding that the PFVc was specifically directed to fuse with the second pair of ISVs promoted us to explore the underlying molecular mechanisms. Previous studies have revealed many molecules that guide vessel extension by regulating the vascular microenvironment or arterial-venous identification. Chemokines guide cell migration with gradients, among which Cxcl12 and Cxcl4 ligand-receptor pairs are essential for guiding endothelial migration.^3^ In our initial study, single-cell transcriptome analysis of zebrafish embryonic endothelial cells at the larval stage revealed that *cxcr3.3*, *cxcr4a*, *cxcr4b*, and *cxcr7b* were expressed in vascular endothelial cells, suggesting their critical roles in regulating vascular development (Figure 3A through 3E′; Figures S5 and S6). Moreover, *cxcr4a* had a much higher expression level than *cxcr3.3*, *cxcr4b*, and *cxcr7b* in arterial endothelial cells, further stressing its importance in these cells (Figure 3B through 3E′). Whole-mount in situ hybridization results consistently confirmed that *cxcr4a* was specifically highly expressed in the developing CBVL (Figure 3F through 3F″). Aiming to further confirm that the directional migration of PFVc is related to *cxcr4a*, we injected AMD3100, which could competitively bind to Cxcr4a in the embryos at 48 hpf. As a result, the PFVc filopodium of treated embryos failed to reach the second pair of ISVs and eventually anastomosed with DA instead (with a ratio of ≈55%), with blood flow from DA to PFVc (Figure S7; Video S4). Furthermore, *cxcr4a* knockdown and *cxcr4a* knockout were performed. *Cxcr4a* knockdown also caused similar phenotypes with misguided PFVc (with a ratio of ≈60%). In comparison, *cxcr4a* knockout caused a more serious phenotype with the loss of PFVc (with a ratio of ≈90%; Figure 3G through 3K).

**Figure 4.**
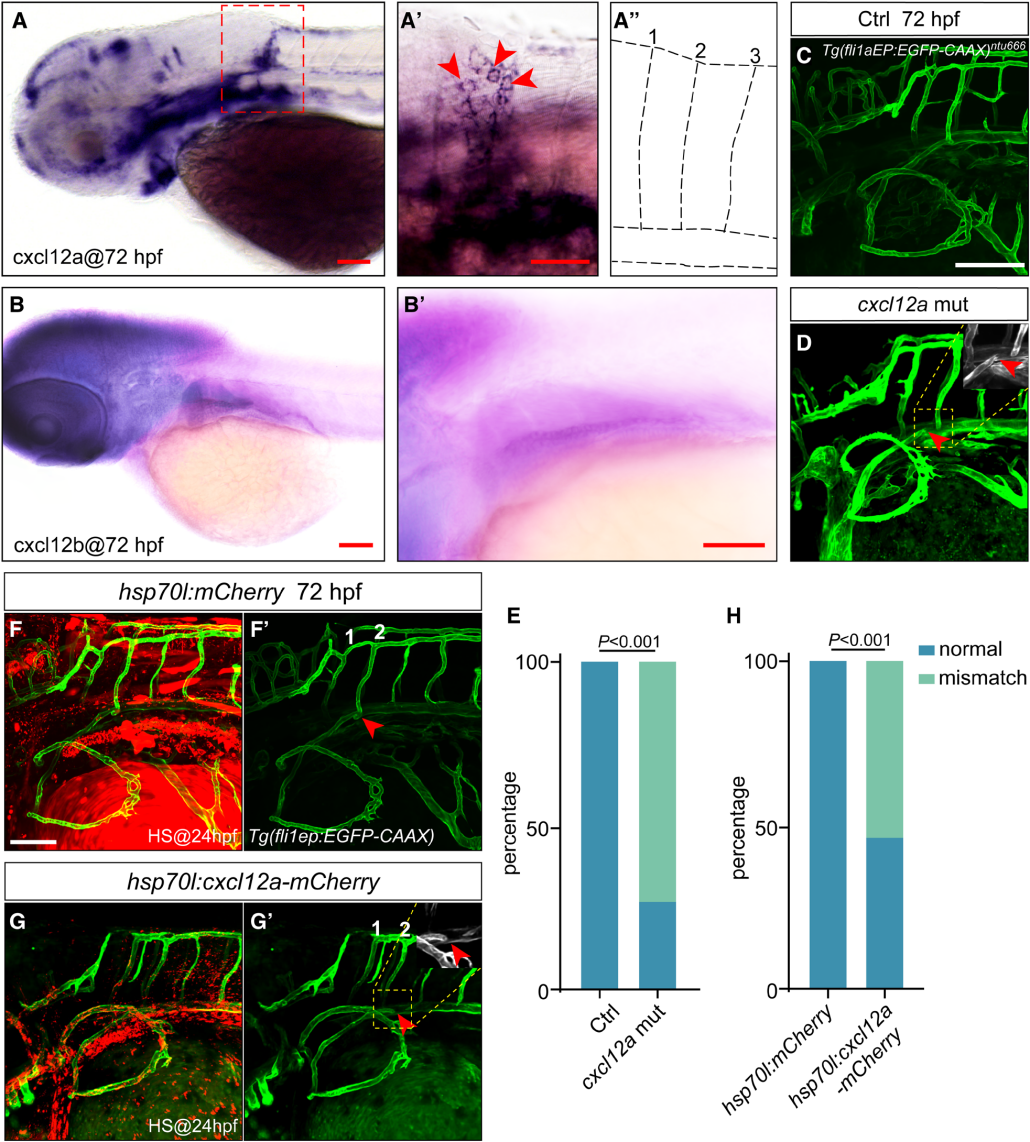
**Cxcl12a (C-X-C motif chemokine ligand 12a) is the ligand of Cxcr4a (C-X-C motif chemokine receptor 4a) in the regulation of anastomosis of pectoral fin vessel c (PFVc)–intersegmental vessels (ISVs). A** through **A″**, Whole-mount in situ hybridization (WISH) analysis of *cxcl12a* expression at 72 hours post-fertilization (hpf). **A′**, cxcl12a positive cells (marked with red arrowheads) were around the second pair of ISVs. **A″**, The schematic illustration of the first 3 pairs of ISVs. **B** and **B′**, WISH analysis of *cxcl12b* expression at 72 hpf. **C**, Live-imaging analysis of the long-distance anastomosis of PFVc-ISVs on *Tg(fli1aEP:EGFP-CAAX*)^*ntu666*^ transgenic zebrafish. **D**, Knockout of *cxcl12a* led to the misconnection of PFVc-ISVs. **E**, The ratio of misconnection caused by knockout of *cxcl12a*. The experiments were repeated 4×; 5 embryos were used in control and *cxcl12a* mutants for each experiment. **F** and **F′**, Injection of *hsp70l:mCherry* construct did not affect the anastomosis of PFVc-ISVs. **G** and **G′**, Overexpression of *cxcl12a* led to the misconnection of PFVc-ISVs. **H**, The ratio of misconnection caused by overexpression of *cxcl12a*. The experiments were repeated 4×; 5 embryos were used in *hsp70l:mCherry* and *hsp70l:cxcl12a-mCherry* groups for each experiment. HS@24hpf indicates heatshock at 24 hpf. Scale bars=100 µm.

Because *cxcr4a* has 2 ligands in zebrafish, *cxcl12a* and *cxcl12b*, we examined the expression pattern of *cxcl12a* and *cxcl12b*. As expected, the result demonstrated that *cxcl12a* was highly expressed in the area around the second pair of ISVs but not the other ISVs at 72 hpf (Figure 4A through 4A″), while the *cxcl12b* was expressed in other different areas (Figure 4B through 4B′). Therefore, we speculated that Cxcl12a guided the directional migration and anastomosis of PFVc to the second pair of ISVs via interacting with Cxcr4a. The subsequent experiments showed that *cxcl12a* mutation caused the misguided migration and fusion of PFVc (with a ratio of ≈70%; Figure 4C through 4E). Consistently, overexpression of *cxcl12a* in larger areas of the embryos also resulted in the misconnection between PFVc and its target ISVs (with a ratio of ≈55%; Figure 4F through 4H).

### Cxcl12a Originated From Monocyte Regulates PFVc Growth and Anastomosis

**Figure 5.**
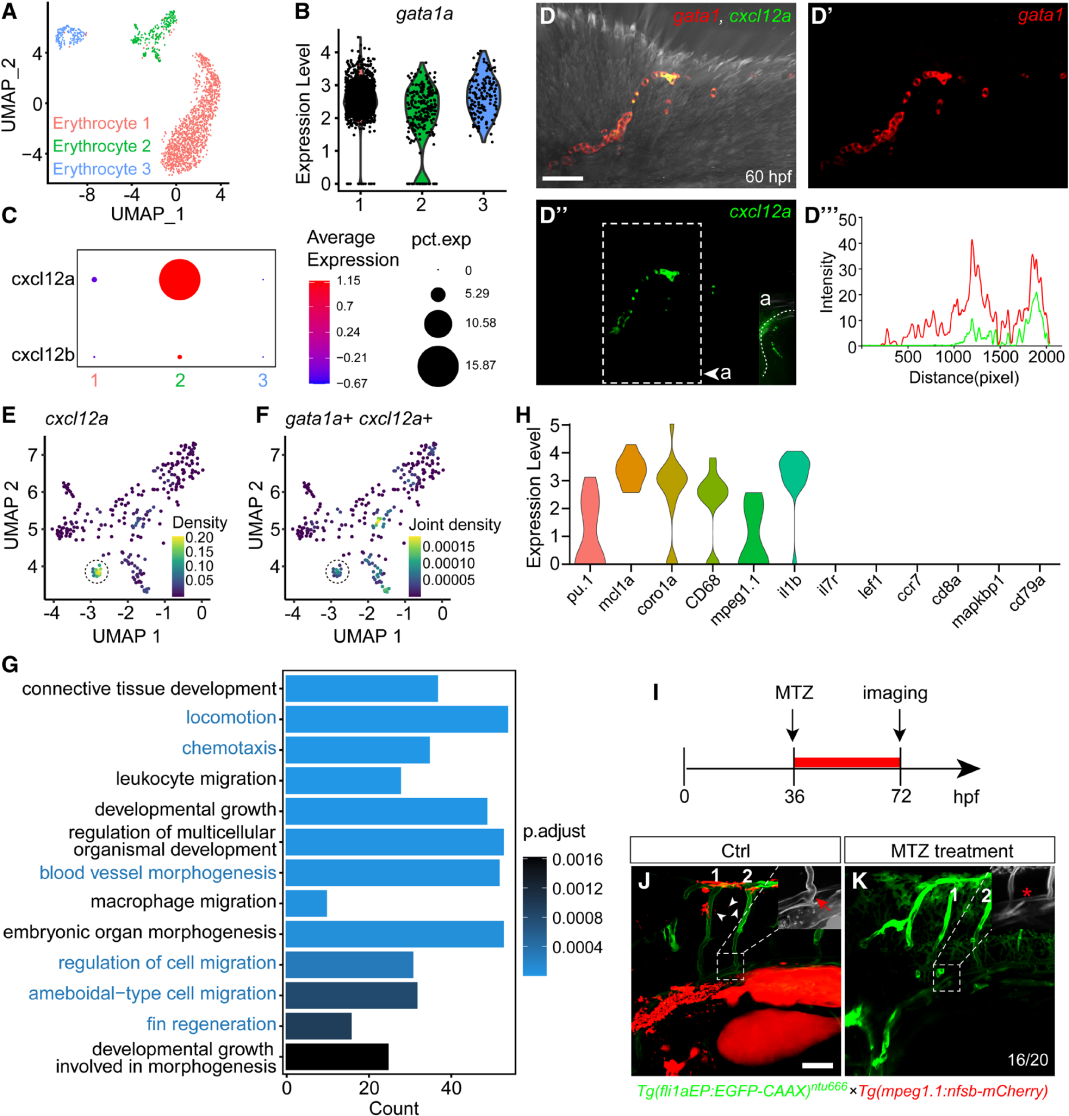
**Monocyte-secreted cxcl12a regulated the pectoral fin vessel c (PFVc)–intersegmental vessel (ISV) anastomosis. A**, Clustering result of myeloid cells (data from GSE178150) by Uniform Manifold Approximation and Projection (UMAP). **B**, Violin plot showing the expression of *gata1a* in the 3 clusters in **A**. **C**, Dot plot showing the average expression level and cell expression ratio of *cxcl12a* in the 3 clusters in **A**. **D** through **D″**, Fluorescent in situ hybridization (FISH) analysis of *cxcl12a* expression in the second pair of ISVs on *Tg(gata1:DsRed*) transgenic zebrafish embryos. **D″** Side view of *cxcl12a* expression with dashed lines indicating the larva skin. **D‴**, The green and red fluorescence intensity along the second pair of ISVs in **D**. **E** and **F**, Coexpression analysis of *gata1* and *cxcl12a* in the cells of erythrocyte 2 cluster in **A**; dashed circle marked the cells with a high coexpression of *gata1* and *cxcl12a*. **G**, GO analysis of the genes in *gata1* and *cxcl12a* coexpression cells. **H**, Expression of the immune cell marker genes in the *gata1*-*cxcl12a*–expressing cells. **I**, Schematic diagram showing the metronidazole (MTZ)-treated nitroreductase (NTR) depletion experiment on *Tg(fli1aEP:EGFP-CAAX*)^*ntu666*^×*Tg(mpeg1.1:nfsb-mCherry*) double transgenic zebrafish. **J**, Live-imaging analysis of the long-distance anastomosis of PFVc-ISVs in the control (ctrl) cluster at 72 hours post-fertilization (hpf; white arrowheads indicate the mpeg1.1 positive cells and the red arrow indicates the correct anastomosis of PFVc-ISVs). **K**, NTR depletion of mpeg1.1 positive cells led to the misconnection of PFVc-ISVs (marked with asterisks). The experiments were executed in 4 replications; 5 embryos were used in each experiment. pct.exp indicates percent cells within the cluster. Scale bars=50 µm.

**Figure 6.**
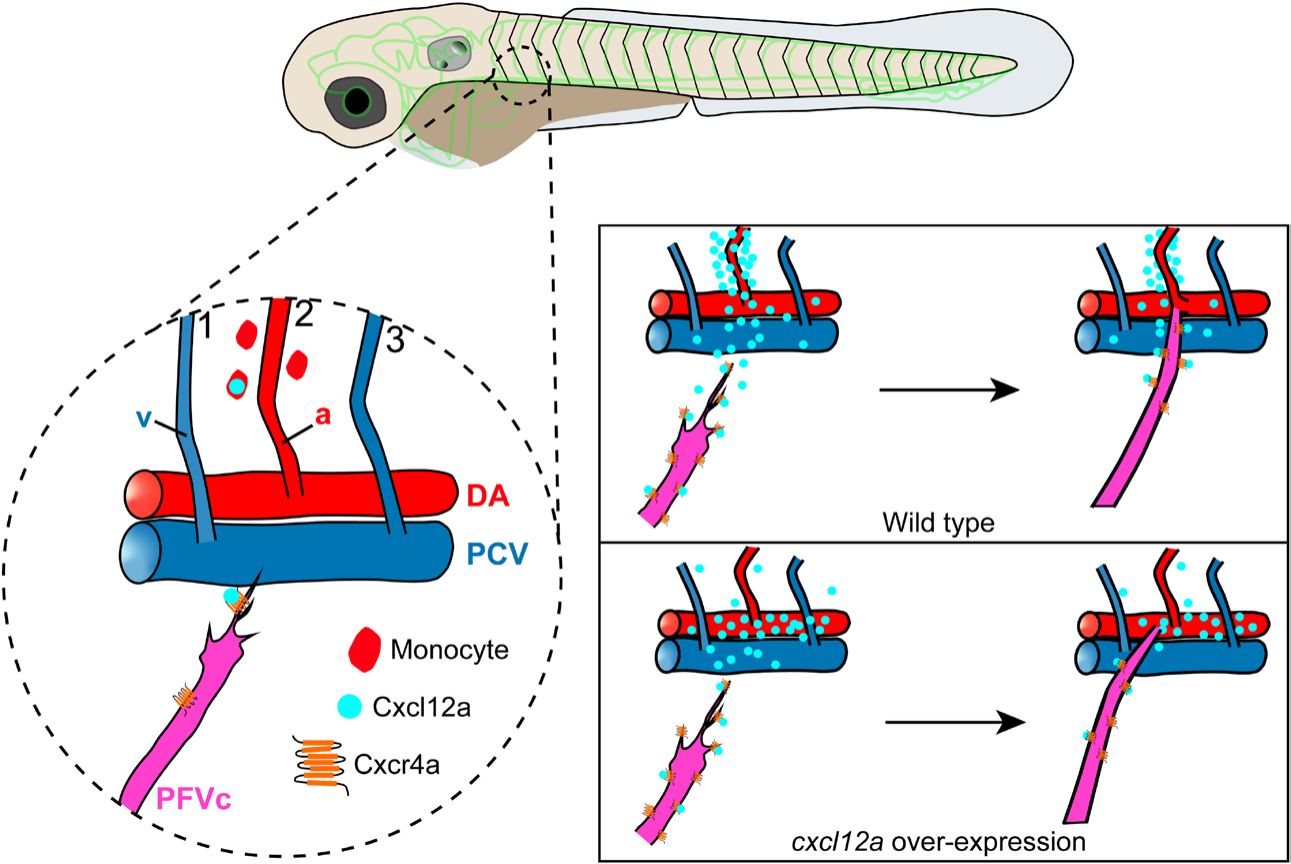
**The proposed molecular mechanism guiding the directed long-distance pectoral fin vessel c (PFVc) migration and anastomosis.** a indicates artery; Cxcl12, C-X-C motif chemokine ligand 12a; Cxcr4a, C-X-C motif chemokine receptor 4a; DA, dorsal aorta; PCV, posterior cardinal vein; and v, vein.

Through whole-mount in situ hybridization, we observed that the cells expressing *cxcl12a* were morphologically similar to monocytes. To better understand these cells, we reanalyzed the single-cell RNA-sequencing data and screened the cells expressing *gata1a*, which is expressed in early myeloid cells. We found 3 distinct clusters (erythrocytes 1–3), among which the erythrocyte 2 cluster had the highest expression level and expression ratio of *cxcl12a* (Figure 5A through 5C). Using fluorescent in situ hybridization, we observed that the cells expressing *cxcl12a*, which were adjacent to the second pair of ISVs, were gata1 positive (Figure 5D through 5D‴). Subsequent analysis of the *cxcl12a* and *gata1a* coexpressing cells revealed a subset of cells within the cluster of erythrocyte 2, which was identified as displaying relatively higher levels of coexpression of *gata1a* and *cxcl12a* (Figure 5E and 5F). GO analysis showed that the genes in *gata1a-cxcl12a* positive cells were involved in locomotion, chemotaxis, blood vessel morphogenesis, and cell migration regulation, consistent with our observations (Figure 5G). Additionally, the marker genes *pu.1*, *coro1a*, *si:ch211-212k18.7 (CD68*), *il1b*, and *mpeg1.1*, which are indicative of monocyte and macrophage presence, were highly expressed in the subset of *gata1a-cxcl12a* coexpressing cells, suggesting that these cells may represent monocytes (Figure 5H). To functionally prove this finding, we performed the NTR depletion of macrophage in the embryos and subsequently observed the misconnection of PFVc to DA as well (Figure 5I through 5K; Videos S5 and S6). These findings indicate that a subset of monocytes plays a crucial role in facilitating directed long-distance vascular migration and anastomosis through the secretion of Cxcl12a (Figure 6).

## Discussion

Organotypic vasculature is essential for organs as it provides oxygen, nutrients, and signaling molecules to ensure the proper development and function of the organs. The process of organotypic vascular formation needs to be tightly coordinated with its host organ development. Although previous studies have observed the formation of the circumferential blood loop, which separates the pectoral fin into the apical fold region and the endoskeletal region, the details of the pectoral fin vascular development remain unclear.^24,26^

In this study, we elucidated the intricate molecular and cellular mechanisms underlying the anastomosis of the fin artery. Meanwhile, the PFVc extended and connected to the second pair of arterial ISVs as an artery, consistent with the previous report,^27^ allowing the arterial blood to move from the DA into the PFVs and finally go to the common cardinal vein. In this respect, a question arises as to why the PFVc must establish a connection with the ISVs rather than directly with the DA. We infer that the PFVc prefers connecting to the ISVs due to their relatively equivalent vessel caliber and blood flow.

One of the fundamental questions during vascular formation is when and how arteries and veins are specified. At the beginning, the specification of the endothelial cells was proposed to be impacted by the blood flow in the vessels.^28–31^ Then, studies have found that the specification began at the budding of tip cells based on genetic mechanisms before blood circulation.^32–34^ So far, few studies have been reported to elucidate the specification of organotypic vessels. In the regenerated fin, the vascularization was found to initiate from venous cells in the stump, generating new arteries and vascular plexus, which is regulated by Notch signaling.^35^ Recently, another study has described the process of pectoral fin vascular formation in zebrafish^27^; however, the molecular guiding cues and mechanisms involved in directing the growth and migration of the vessels remain unclear. Here, our findings demonstrated that at the early stage of sprouting before the anastomosis and perfusion of the vessels, the artery and vein have differentiated via the activation of Notch signaling–directed endothelial tip cells and regulation of *flt1* expression, implying that the CBVL specification was determined genetically, which is consistent with previous study.^29^

There are 2 kinds of vascular anastomosis during the pectoral fin development. Lenard et al^36^ reported the cranial vasculature process of zebrafish embryo and mentioned the 2 typical fusion features, including the anastomosis of palatocerebral artery and palatocerebral artery–communicating vessel. In our study, the anastomosis of PFVc-ISV was mediated by new sprouting with another preexisting vessel. Interestingly, the question of how the PFVc could specifically reach and fuse with the second pair of ISVs in such a long distance attracted our attention and prompted us to explore the mechanisms involved in this process. Considering that previous studies have shown that the Cxcl12a/Cxcr4a axis mediates vascular patterns,^37–39^ we, therefore, speculated that the anastomosis between PFVc with the target ISVs might also be mediated by Cxcl12a/Cxcr4a signaling. As expected, our experiments showed highly specific expressions of *cxcr4a* and *cxcl12a* in the PFVc and around the second pair of ISVs, respectively. Besides, either binding blocking or overexpression of *cxcl12a* in other segments of zebrafish both misguided the PFVc growth, leading to further extension and fusion with other untargeted vessels (DA). Altogether, we speculate that the *cxcl12a* is expressed and distributed in gradients around the second pair of ISVs. It can bind to cxcr4a expressed on the surface of PFVc and guide the filopodium elongation from PFVc to the target ISVs. The results proved that the Cxcl12a/Cxcr4a axis was essential in mediating the long distance–specific migration and anastomosis of PFVc-ISV.

Although the role of Cxcl12a in artery development has been proved in our research and previous studies,^13^ the specific cellular sources responsible for its expression during this process remain unidentified. Our study has unveiled, for the first time, that a subset of monocytes containing a proportion of naive macrophages may be implicated in the expression of *cxcl12a*. Furthermore, a recent study utilizing single-cell analysis has identified macrophages with proangiogenic activities that interact with vascular endothelial cells in various organs during prenatal development, which is in line with our result.^40^

In summary, our study investigated whether the arterial-venous specification of the PFVs was predetermined genetically. More importantly, our study revealed the specific long-distance migration and anastomosis between the PFV and the second pair of the ISVs and further proved that this process was mediated by the Cxcl12a/Cxcr4a axis. During this process, the *cxcl12a* was specifically expressed by monocytes. The findings provide new insights into the developmental process and molecular mechanisms of vascular formation in zebrafish.

## Article Information

### Sources of Funding

This study was supported by grants from the National Natural Science Foundation of China (92368104 received by D. Liu; http://www.nsfc.gov.cn), Natural Science Foundation for colleges and universities in Jiangsu Province (19KJB180022 received by X. Duan), and Science and Technology Foundation of Nantong City (JC22022019 received by X. Lu; http://kjj.nantong.gov.cn/).

### Disclosures

None.

### Supplemental Material

Figures S1–S7

Videos S1–S6

Major Resources Table
